# C-reactive protein isoforms as prognostic markers of COVID-19 severity

**DOI:** 10.3389/fimmu.2022.1105343

**Published:** 2023-01-18

**Authors:** Blanca Molins, Marc Figueras-Roca, Oliver Valero, Víctor Llorenç, Sara Romero-Vázquez, Oriol Sibila, Alfredo Adán, Carolina García-Vidal, Alex Soriano

**Affiliations:** ^1^ Group of Ocular Inflammation: Clinical and Experimental Studies, Institut d’Investigacions Biomèdiques Agustí Pi i Sunyer (IDIBAPS), Barcelona, Spain; ^2^ Institut Clínic d’Oftalmologia (ICOF), Hospital Clínic, Barcelona, Spain; ^3^ Statistical Department, Universitat Autònoma de Barcelona, Barcelona, Spain; ^4^ Respiratory Department, Hospital Clinic of Barcelona-IDIBAPS, CIBERES, University of Barcelona, Barcelona, Spain; ^5^ Department of Infectious Diseases, Hospital Clinic of Barcelona-IDIBAPS, University of Barcelona, Barcelona, Spain; ^6^ CIBERINF, Barcelona, Spain

**Keywords:** C-reactive protein, COVID-19, isoforms, inflammation, prognosis

## Abstract

C-reactive protein (CRP), an active regulator of the innate immune system, has been related to COVID-19 severity. CRP is a dynamic protein undergoing conformational changes upon activation in inflammatory microenvironments between pentameric and monomeric isoforms. Although pentameric CRP is the circulating isoform routinely tested for clinical purposes, monomeric CRP shows more proinflammatory properties. Therefore, we aimed to determine the potential of monomeric CRP in serum as a biomarker of disease severity in COVID-19 patients (admission to intensive care unit [ICU] and/or in-hospital mortality). We retrospectively determined clinical and biological features as well as pentameric and monomeric CRP levels in a cohort of 97 COVID-19 patients within 72h of hospital admission. Patients with severe disease had higher levels of both pentameric and monomeric CRP. However, multivariate analysis showed increased mCRP but not pCRP to be independently associated to disease severity. Notably, mCRP levels higher than 4000 ng/mL (OR: 4.551, 95% CI: 1.329-15.58), together with number of co-morbidities, low lymphocyte count, and procalcitonin levels were independent predictors of disease severity in the multivariate model. Our results show the potential of mCRP levels as a marker of clinical severity in COVID-19 disease.

## 1 Introduction

Coronavirus disease 2019 (COVID-19), caused by severe acute respiratory syndrome coronavirus 2 (SARS-CoV-2) infection was declared in March 2020 a global pandemic by the World Health Organization with more than 4 million related deaths since then (1). COVID-19 is asymptomatic or mild in most cases. Yet, 10% of patients present severe disease due to pneumonia, acute respiratory distress syndrome (ARDS), systemic inflammation, thrombosis, and multiorgan failure, causing death in 1-3% of patients. Indeed, some patients with mild symptoms may present a sudden progression to severe disease (2).

Noteworthy, although hospitalization for COVID-19 is needed only in a minority of patients and disease management has majorly improved since the first outbreak in March 2020, the highly infectious nature of SARS-CoV-2 driving to high incidence spikes in short periods of time is still posing a threat to health care systems worldwide. Thus, predicting individual prognosis is crucial to provide a personalized treatment to reduce mortality. Age, male sex, hypertension, the presence of certain co-morbidities, and several blood biomarkers have been consistently associated with worse prognosis (3, 4).

C-reactive protein (CRP), an acute phase reactant and an active regulator of the innate immune system, is increased in COVID-19 patients and has been associated to disease severity (5, 6). Among its multiple functions, CRP activates the classical pathway and inactivates the alternative pathway of the complement system (7). CRP is mainly synthesized in the liver upon interleukin- (IL-) 6 induction and its levels can increase up to 100-fold in response to several forms of tissue damage, infection and inflammation. In plasma, CRP circulates as a disk-shaped pentamer (also known as pentameric CRP, pCRP) composed of five 23 kDa non-covalently bound subunits (8). Nevertheless, low pH, oxidative stress and bioactive lipids from activated cells can dissociate pCRP into its monomeric subunits (9, 10) by means of phospholipase A_2_ activation and subsequent lysophosphatidylcholine cell surface exposure (11). This conformation of CRP, named monomeric CRP (mCRP), shows different biological functions and antigenicity-expressing neoepitopes than pCRP representing the tissue-associated insoluble form of CRP. Although there is no standardized method, circulating levels of mCRP in serum can be measured by means of customized ELISA (12). Unlike pCRP, mCRP presents a prothrombotic and proinflammatory phenotype (13–16). Given that most reports on the role of CRP in systemic diseases are based on the pentameric conformation, additional research on the specific implications of mCRP over pCRP should be addressed.

Considering the hyperinflammatory nature of COVID-19 complications and the fact that mCRP dissociates from pCRP in proinflammatory microenvironments and it is also the main active proinflammatory isoform of CRP, we hypothesized that mCRP could be considered as a more specific prognostic marker of inflammatory progression and severity than pCRP in COVID-19. Thus, we aimed to evaluate mCRP and pCRP levels in COVID-19 patients at hospital admission to determine their prognostic value to progression to more severe disease forms.

## 2 Methods

### 2.1 Study design, patients, and data collection

We performed an observational retrospective study of 97 patients admitted to the hospital (Hospital Clinic of Barcelona, Spain) for > 48h with confirmed acute SARS-CoV-2 infection by rRT-PCR performed on nasopharyngeal and throat swabs between March 1st and September 30th 2020. Included patients had serum samples preserved in the COVIDBANK within 72h of admission for further determination of circulating levels of mCRP. The COVIDBANK is a biorepository of biological samples from SARS-CoV-2 patients treated in Hospital Clinic of Barcelona with the aim of providing samples of quality for SARS-COV-2-related scientific research. The Institutional Ethics Committee of Hospital Clinic of Barcelona approved the study (HCB/2020/0874) and patients gave their informed consent to the COVIDBANK.

Data were retrospectively obtained for all patients included in the study from the electronic health records. Variables included were age, sex, and co-morbidities (chronic heart disease, diabetes mellitus, chronic kidney disease, hypertension, solid malignant neoplasm, chronic respiratory disease, haematologic disease, hepatopathy, solid organ transplant, HIV). Biochemical variables included blood cell count (eosinophils, basophils, lymphocytes), creatinine, D-dimer, ferritin, lactate dehydrogenase (LDH), procalcitonin, troponin, and pCRP at admission. The composite outcome variable included the need of intensive care unit (ICU) admission and/or in-hospital mortality at any time during in-hospital stay. In addition, mCRP levels were determined from preserved serum samples of the COVIDBANK.

### 2.2 Serum mCRP determination

Serum mCRP was detected with the ELISA protocol described by Zhang et al. with some modifications (12, 17). Briefly, mouse anti-human CRP mAb CRP-8 (Sigma-Aldrich, C1688) was immobilized as capture antibody at 1:1000 in coating buffer (10 mM sodium carbonate/bicarbonate, pH 9.6) for 18h at 4°C. This commercially available monoclonal antibody has been reported to specifically recognize mCRP, but not pCRP (18). After washing three times for 2 min each with TBS, non-specific binding was blocked with filtered 1% BSA-TBS for 1 hour at 37°C. Samples were diluted 1:100 in blocking buffer and added into wells for 1h at 37°C. Then, washing step was repeated and samples were incubated with sheep anti-human CRP polyclonal antibody (MBS223280, MyBioSource) at 1:5000 in blocking buffer for 1h at room temperature (RT), before incubation with a HRP-labelled donkey anti-sheep IgG (Abcam) at 1:10000 in blocking buffer for 30 min at RT. Signalling was detected with VersaMax Microplate Reader and the OD value of each sample was calculated as OD_450_–OD_570_ nm. A standard curve was prepared by serial dilutions of mCRP (0-50 ng/mL) obtained by urea-chelation of pCRP (Calbiochem) (16) in blocking buffer (1% BSA-TBS) in the presence of reference diluted sera (1:100). The concentration of mCRP in the reference sera was below detection level and therefore undetectable following 1:100 dilution. Controls using purified pCRP at a concentration of 100 ng/mL, which would be equivalent to 10 µg/mL after 1:100 dilution, only generated background signal, showing specificity for mCRP.

### 2.3 Statistical analysis

Categorical variables were described as absolute (N) and relative (%) frequencies and Chi square or Fisher’s test was used for comparisons. Quantitative variables following a normal distribution were represented as mean ± standard deviation and differences between groups were determined with Student’s t-test. Variables with a non-normal distribution were expressed as median and minimum and maximum value and the Mann-Whitney test was employed to determine significant differences. Linear relationships were analysed by determination of Spearman correlation coefficient. Statistical assumptions were made based on significance level below 0.05.

To determine the variables associated with disease severity (need for ICU admission and/or in-hospital mortality), a multivariable logistic regression model was performed adding the variables with a P value lower than 0.2 in the bivariate analysis. Variables with more than 10% of missing values were excluded. Quantitative variables added to the multivariate analysis were dichotomized as follows: age (>70), pCRP (>10 µg/mL), mCRP (>4000 ng/mL), creatinine (>1.1 mg/dL in women and >1.3 mg/dL in men), D-dimer (>500 ng/mL), lymphocytes (<0.004 x10^9^/L), procalcitonin (>0.5 ng/mL), ferritin (>306 ng/mL in women and >336 ng/mL in men), troponin (>50 ng/mL). Cut-off values of pCRP, creatinine, D-dimer, lymphocyte count, procalcitonin, and ferritin were chosen based on their abnormal levels associated to pathology. The final model was reached through backward stepwise removal of variables with p-value higher than 0.1 and using Wald tests to demonstrate that each model was better than its previous iteration. Odds ratio (OR) with 95% confidence interval (CI) were calculated. To assess the predictive ability of each model, we calculated the area under the receiver operating characteristic (ROC) curve with its respective 95% confidence interval (95% CI) and determined the cut-off value to maximize sensitivity and specificity. Statistical analyses were performed using SAS v9.4 software (SAS Institute Inc., Cary, NC, USA).

## 3 Results

Study population consisted of 97 patients admitted to Hospital Clínic of Barcelona between March and September 2020 for > 48h with confirmed acute SARS-CoV-2 infection. The mean age was 60 ± 17 years and 59 (61%) were males. The most common co-morbidities were hypertension (50,5%), diabetes mellitus (28.9%), chronic heart disease (23.7%), chronic respiratory disease (21.6%), solid malignant neoplasm (15.5%), and chronic kidney disease (15.5%). Other minor co-morbidities included chronic liver disease, hematologic disease, solid organ transplant, and HIV infection. A total of 27 (27.8%) patients were admitted to the ICU and the in-hospital mortality rate was 25.8% (**Table 1**). Although the majority of patients that died were previously admitted to ICU (15/25, 60%), some died in normal ward (10/25, 40%). Severe disease was defined as ICU admission and/or in-hospital mortality.

**Table 1:** Clinical and analytical features of COVID-19 patients upon admission and significant risk factors for disease severity (ICU admission and/or in-hospital mortality) on bivariate analysis.

Variable	Total N=97	Non-severe disease N=60	Severe disease N=37	P-value
Age, mean (SD), years	60.3 (16.9)	55.02 (16.1)	68.76 (14.8)	*<0.001
Male, N (%)	59 (60.2)	36 (61.0)	23 (39.0)	0.911
Pre-existing comorbidities, N (%)				
Chronic heart disease	23 (23.7)	7 (30.4)	16 (69.6)	*<0.001
Diabetes mellitus	28 (28.9)	15 (53.6)	13 (46.4)	0.285
Chronic kidney disease	15 (15.5)	3 (20.0)	12 (80.0)	*<0.001
Hypertension	49 (50.5)	24 (49.0)	25 (51.0)	*0.008
Solid malignant neoplasm	15 (15.5)	5 (33.3)	10 (66.7)	*0.013
Chronic respiratory disease	21 (21.6)	15 (71.4)	6 (28.6)	0.308
Other pathologies		3 (33.3)	6 (66.7)	0.064
Haematologic disease	2 (2.1)	1 (50.0)	1 (50.0)	1.000
Chronic liver disease	3 (3.1)	0 (0)	3 (100)	0.053
Solid organ transplant	3 (3.1)	1 (33.3)	2 (66.7)	0.556
HIV	3 (3.1)	1 (33.3)	2 (66.7)	0.556
Number of pathologies, median (min-max)	1.67 (0.00-5.00)	1.20 (0.00-5.00)	2.43 (0.00-5.00)	*<0.001
Analytical variables, median (min-max)				
Basophils, 10^9^/L	0.00 (0.00-0.10)	0.00 (0.00-0.10)	0.00 (0.00-0.10)	0.838
Eosinophils, 10^9^/L	0.00 (0.00-0.50)	0.00 (0.00-0.40)	0.00 (0.00-0.50)	0.150
Lymphocytes, 10^9^/L	1.00 (0.10-2.90)	1.15 (0.30-2.20)	0.60 (0.10-2.90)	*<0.001
Creatinine, mg/dL	0.87 (0.24-6.49)	0.85 (0.50-4.05)	1.17 (0.24-6.49)	*0.012
D-dimer, ng/mL	650 (200-28400)	450 (200-6000)	1200 (400-28400)	*<0.001
Ferritin, ng/mL	596 (12-6126)	487 (12-4309)	740 (21-6126)	0.127
LDH, U/L	302 (148-971)	298 (148-675)	332 (168-971)	0.178
Procalcitonin, ng/mL	0.12 (0.00-15.03)	0.08 (0.00-1.58)	0.18 (0.03-15.03)	*<0.001
Troponin, ng/L	7.20 (0.00-803)	3.25 (0.00-803)	11.65 (0.00-304)	*<0.001
pCRP, µg/mL	6.43 (0.00-33.50)	5.63 (0.00-33.34)	11.61 (1.65-33.50)	*0.013
mCRP, ng/mL	1860 (30-9805)	206.0 (30-8086)	3551.3 (30-9806)	*<0.001
Log, mCRP/pCRP	5.44 (-0.11-7.82)	4.75 (-0.11-7.82)	5.64 (0.54-7.27)	0.216

*Statistically significant.

**Figure 1:**
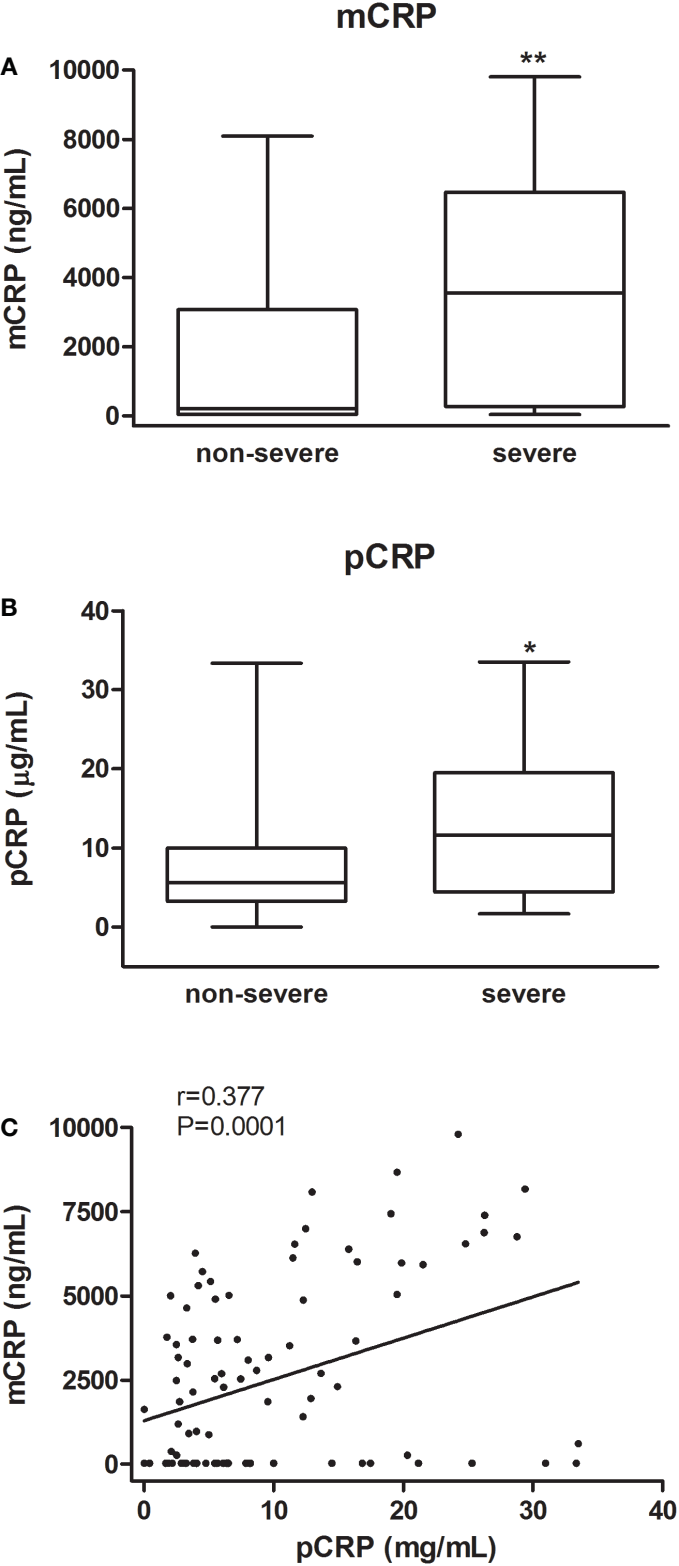
mCRP and pCRP levels in COVID-19 patients. Box-and whisker plots of mCRP **(A)** and pCRP **(B)** levels according to disease severity (ICU-admission and/or in-hospital mortality). Median values are highlighted by solid lines. Statistical analysis was conducted using Mann-Whitney test (*P < 0.05, **P < 0.001). Correlation between mCRP and pCRP **(C)**.

Patients with severe disease (37/97) were older (68.76 ± 14.8 vs. 55.02 ± 16.1 years, P<0.001) and had more co-morbidities (2.43 [0.00-5.00] vs. 1.20 [0.00-5.00], P<0.001). Patients with higher levels at admission of creatinine (1.17 [0.24-6.49] vs. 0.85 [0.50-4.05] mg/dL, P=0.012), D-dimer (1200 [400-28400] vs. 450 [200-6000] ng/mL, P<0.001), procalcitonin (0.18 [0.03-15.03] vs. 0.08 [0.00-1.58] ng/mL, P<0.001), troponin (11.65 [0.00-304] vs. 3.25 [0-803] ng/L, P<0.001), pCRP (11.61 [1.65-33.50] vs. 5.63 [0.00-33.34] µg/mL, P=0.013), and mCRP (3551.3 [30-9806] vs. 206 [30-8086], ng/mL P<0.001) and lower lymphocyte count (0.60 [0.10-2.90] vs. 1.15 [0.30-2.20] 10^9^/L, P<0.001) were also more likely to develop severe disease. Chronic kidney and heart disease, hypertension, and solid malignant neoplasm were also associated to disease severity (**Table 1**). As shown in **Figure 1**, patients that required ICU admission and/or died had higher levels of both monomeric (*P<0.001) and pentameric CRP (*P=0.013). mCRP significantly correlated with pCRP (r=0.377, P<0.001), although the correlation coefficient was only intermediate (**Figure 1C**). When analysing the ratio log_mCRP/pCRP no association with disease severity was found. In fact, when determining the correlation of log_mCRP/pCRP with established severity markers only a statistically significant correlation was observed for creatinine (r=0.241, P=0.019) and troponin (r=0.264, P=0.015), but not for procalcitonin (r=-0.007, P=0.94), lymphocyte count (r=-0.173, P=0.096), ferritin (r=-0.157, P=0.139), and D-dimer (r=0.133, P=0.208). Instead, mCRP significantly correlated with more markers of severity including procalcitonin (r=0.278, P=0.007), lymphocyte count (r=-0.335, P=0.001), creatinine (r=0.205, P=0.044), troponin (r=0.275, P=0.01), and D-dimer (r=0.329, P=0.001) but not with ferritin (r=0.087, P=0.410). Nevertheless, in most cases correlation coefficient was weak (r<0.3).

**Figure 2:**
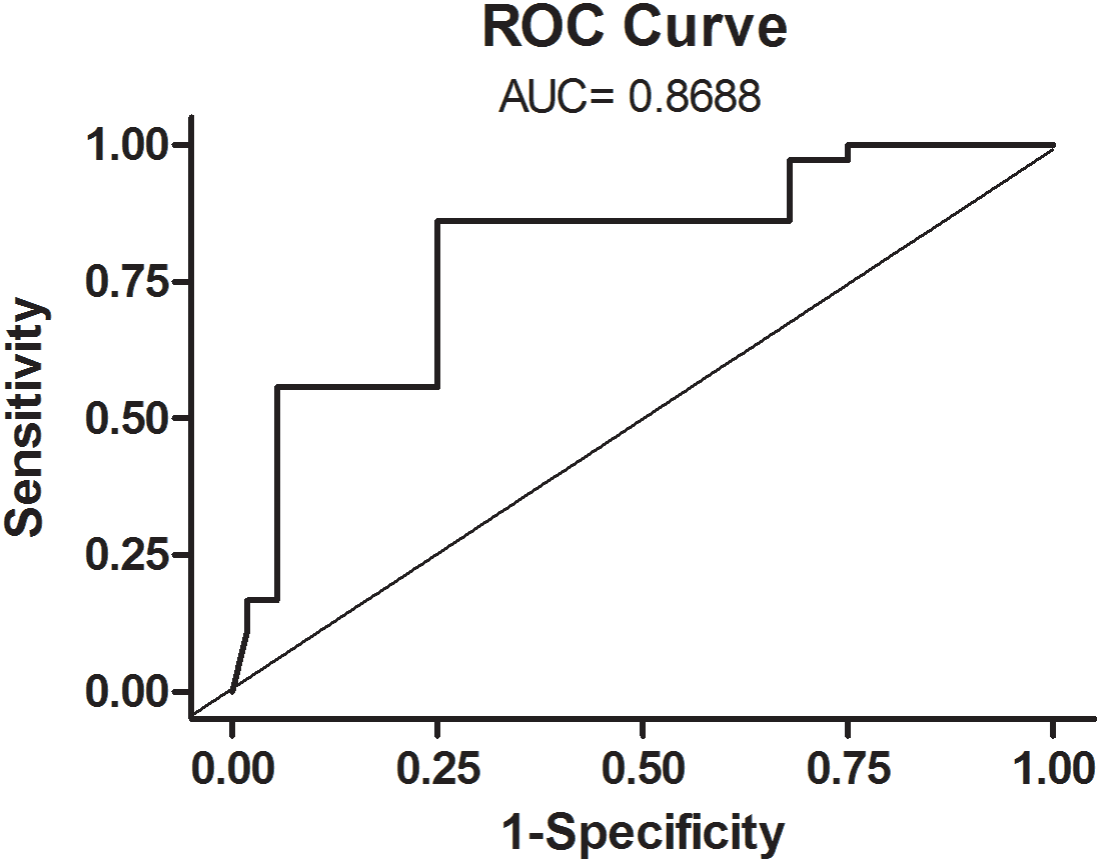
ROC curve of the multivariate model.

Quantitative variables were then dichotomized and added to the multivariate analysis: number of pathologies, high levels of mCRP (>4000 ng/mL) and procalcitonin (>0.5 ng/mL), and low lymphocyte count (<0.004 x10^9^/L) were independently associated to disease severity. Lymphocyte count (OR: 4.615, 95% CI: 1.493-14.26), procalcitonin (OR: 18.199, 95% CI: 2.235-148.17), and mCRP (OR: 4.551, 95% CI: 1.329-15.58) were retained in the model as independent predictors of severity (**Table 2**). The area under the ROC curve (AUC) was 0.869 (95% CI: 0.794-0.945, and the best cut-off had a 86.1% sensitivity and 75.0% specificity) showing a good ability to predict in-hospital mortality (**Figure 2**).

**Table 2:** Significant risk factors for disease severity (ICU admission and/or in-hospital mortality) on multivariate regression analysis.

Variable	OR	95% CI	P- value
Number of pathologies			*0.036
1 vs. 0	10.660	1.448-78.48	
2 vs. 0	12.135	1.787-82.41	
2 vs. 1	1.138	0.344-3.77	
mCRP >4000 ng/mL	4.551	1.329-15.58	*0.016
Lymphocytes <0.004x10^9^/L	4.615	1.493-14.26	*0.008
Procalcitonin >0.5 ng/mL	18.199	2.235-148.17	*0.007

*Statistically significant.

## 4 Discussion

Since the first outbreak in early 2020 huge labour has been devoted to understand COVID-19 pathophysiology and to identify prognosis factors for disease severity worldwide. The wide range of signs, symptoms and clinic severity urge to a personalized approach for disease management. In this regard, certain blood biomarkers such as IL-6, ferritin, D-dimer, and CRP may be able to anticipate the development of the cytokine storm leading to COVID-19 complications (19, 20). Here, we provide insights on the potential of the conformational isoforms of CRP to predict disease severity, defined as ICU admission and/or in-hospital mortality. Our results show that high levels of circulating mCRP in COVID-19 patients at hospital admission are independently associated to disease severity.

CRP is a dynamic protein subjected to conformational changes upon activation in inflammatory microenvironments between circulating (pCRP) and monomeric (mCRP) tissue-based isoforms. Notably, mCRP manifests potent proinflammatory effects and activates platelets (16), leukocytes (15), and endothelial cells (21). Moreover, mCRP deposition has been localized in inflamed tissues, thus suggesting an active role in the progression of several inflammatory disorders including Alzheimer’disease (22), cardiovascular disease (23), and age-related macular degeneration (24). Although mCRP could represent a more accurate marker of inflammation, pentameric pCRP is the CRP isoform determined for clinical purposes.

Currently, there are no commercially available assays to determine serum or plasma levels of mCRP, likely due to the insoluble nature of the monomeric subunit. However, several reports have developed customized ELISA assays to determine circulating mCRP using specific antibodies against mCRP. Some authors have generated their own antibodies against mCRP (25–27), others have used mCRP antibodies (clone 8C10) developed by Potempa et al. (28), while others have used the commercially available clone CRP-8 that has been described to specifically recognize mCRP (12, 29). In our study, we followed the protocol described by Zhang et al., and used the monoclonal antibody CRP-8 to quantify circulating mCRP in our cohort as this protocol avoided cross-reactivity with pCRP. Alternatively, flow cytometry has also been used by some authors to determine circulating mCRP (30, 31).

The precise nature of the mCRP detected by ELISA is unclear as both highly denaturated or globular mCRP forms may be present in serum samples. Given the reduced solubility of mCRP it is conceivable that detected mCRP can be also bound to microparticles. Alternatively, circulating mCRP can be found in a globular form with binding properties similar to the pentameric form as recently described by Williams et al. (29). Indeed, further research regarding the structure and function of physiological circulating mCRP is warranted.

No standardized method for circulating mCRP determination has been established yet and different techniques and protocols may result in different mCRP values, which currently limit the translational application of mCRP in the clinical setting. Yet, few studies have determined circulating mCRP levels in several diseases including skin related autoimmune disorders (0-120 ng/mL) (12), Adult-Onset Still-s disease (0-1000 ng/mL) (25), systemic lupus erythematosus (1-7 ng/mL), antineutrophil cytoplasmic antibody-associated vasculitis (5-20 ng/mL) (28), atherosclerosis (0-50 ng/mL) (27), and chronic pulmonary obstructive disease (600-1000 ng/mL) (26). In these studies, mCRP levels were lower than those for hospitalized COVID-19 patients from our study. As a matter of fact, the study of Karlsson et al. also included a control group of healthy subjects with a mean level of mCRP below 10 ng/mL (28). Although differences may be attributed to the method used to determine mCRP it is conceivable that COVID-19 patients at hospital admission may present increased levels of mCRP compared to other pathologies. Because mCRP dissociates from pCRP, it is also conceivable that high levels of pCRP, as the ones of our cohort, lead to higher levels of mCRP. Indeed, mCRP significantly correlated with pCRP although the correlation coefficient was not particularly strong.

Given the wide range of COVID-19 clinical presentation, severe disease was considered on either ICU admission and/or in-hospital death in order to search for high external validity findings. Accordingly, our results showed that both mCRP and pCRP were significantly associated to disease severity in the bivariate analysis. Interestingly, mCRP, but not pCRP, was independently associated to disease severity in the multivariate analysis. Because mCRP represents the proinflammatory conformation of CRP we also aimed to determine whether the ratio mCRP/pCRP was associated to disease severity. Nevertheless, unlike mCRP, this variable was not associated to disease severity. Notably, the multivariate model including mCRP, number of pathologies, low lymphocyte count, and procalcitonin had an AUC of 0.869, with a 86.1% sensitivity and 75.0% specificity in the best cut-off showing a good ability to predict in-hospital mortality. Although our results suggest that mCRP could serve as a prognostic factor of disease severity in COVID-19, a standardized method for determining circulating mCRP should be established before its clinical application.

Whether mCRP plays a role in the progression of COVID-19 or is merely a marker remains unclear. A recent study showed that mCRP can bind to the SARS-CoV-2 spike receptor binding-domain (RBD) and competes with the binding of the spike RBD to angiotensin-converting enzyme 2 (ACE2) receptor (32). Increased levels of CRP induced by SARS-CoV-2 infection may eventually lead to unrestrained inflammation. Indeed, CRP apheresis can reduce the immune response in COVID-19 patients, as showed in a case series report of seven patients (33). Targeting mCRP in COVID-19 patients could represent a novel therapeutic target. Besides CRP apheresis, approaches aimed at inhibiting either CRP dissociation into mCRP (34, 35) or antibodies against mCRP (36) could represent attractive alternatives to prevent the cytokine storm and hyperinflammatory status associated to COVID-19. Yet, caution should be taken when targeting CRP as the effects of CRP isoforms may differ depending on the infection stage; in early phases of infection CRP acts as a soluble pattern recognition receptor targeting necrotic or damaged tissue, while in late phases of infection, unrestrained levels of CRP may cause hyperinflammation with its subsequent complications. mCRP is mainly generated within inflamed tissues and has a short circulating half-life (21), thus suggesting that circulating mCRP might not play a direct role in autoimmune induction. Yet, it may act as an indicator of local mCRP accumulation and subsequent inflammation. Immunostaining of mCRP in damaged infected tissue could shed light on the role of mCRP in COVID-19 pathophysiology.

In summary, despite certain intrinsic limitations including retrospective and single centre nature together with limited sample size, our study shows for the first time the role of circulating mCRP as a prognostic factor of disease severity in COVID-19 which should be confirmed in prospective multicentric studies. It should be also pointed that our patient cohort was recruited during the first outbreak of the pandemic, before proper treatments and vaccines were available, which may differ with the phenotype of current COVID-19 patients as disease is now better understood and treated. Determination of mCRP levels in vaccinated COVID-19 patients would help to better understand the role of mCRP in COVID-19. Further studies are warranted to elucidate the role of mCRP in the different stages of SARS-COV-2 infection in order to identify novel targets to prevent the eventual progression to cytokine storm.

## Data availability statement

The raw data supporting the conclusions of this article will be made available by the authors, without undue reservation.

## Ethics statement

The studies involving human participants were reviewed and approved by The Institutional Ethics Committee of Hospital Clinic of Barcelona. The patients/participants provided their written informed consent to participate in this study.

## Author contributions

BM, MF-R, CG-V, and AS contributed to the design of the study and experiments. BM, OV, SR-V, MF-R, and VL performed the experiments, data capture, and analysis. BM, MF-R, OS, AA, CG-V, and AS performed the data interpretation. All authors contributed to the article and approved the submitted version.

## References

[1] COVID-19 map - johns Hopkins coronavirus resource center. Available at: https://coronavirus.jhu.edu/map.html (Accessed August 16, 2022).

[2] Wu C Chen X Cai Y Xia J Zhou X Xu S. Risk factors associated with acute respiratory distress syndrome and death in patients with coronavirus disease 2019 pneumonia in wuhan, China. JAMA Intern Med (2020) 180:934–43. doi: 10.1001/JAMAINTERNMED.2020.0994 PMC7070509 32167524

[3] Tian W Jiang W Yao J Nicholson CJ Li RH Sigurslid HH. Predictors of mortality in hospitalized COVID-19 patients: A systematic review and meta-analysis. J Med Virol (2020) 92:1875–83. doi: 10.1002/JMV.26050 PMC7280666 32441789

[4] Grasselli G Zangrillo A Zanella A Antonelli M Cabrini L Castelli A. Baseline characteristics and outcomes of 1591 patients infected with SARS-CoV-2 admitted to ICUs of the Lombardy region, Italy. JAMA (2020) 323:1574–81. doi: 10.1001/JAMA.2020.5394 PMC7136855 32250385

[5] Knight SR Ho A Pius R Buchan I Carson G Drake TM. Risk stratification of patients admitted to hospital with covid-19 using the ISARIC WHO clinical characterisation protocol: development and validation of the 4C mortality score. BMJ (2020) 370:m3339. doi: 10.1136/BMJ.M3339 32907855 PMC7116472

[6] Liu F Li L Da X Wu J Luo D Zhu YS. Prognostic value of interleukin-6, c-reactive protein, and procalcitonin in patients with COVID-19. J Clin Virol (2020) 127:104370. doi: 10.1016/j.jcv.2020.104370 32344321 PMC7194648

[7] Black S Kushner I Samols D. C-reactive protein. J Biol Chem (2004) 279:48487–90. doi: 10.1074/jbc.R400025200 15337754

[8] Volanakis JE. Human c-reactive protein: Expression, structure, and function. Mol Immunol (2001) 38:189–97. doi: 10.1016/S0161-5890(01)00042-6 11532280

[9] Lauer N Mihlan M Hartmann A Schlötzer-Schrehardt U Keilhauer C Scholl HPN. Complement regulation at necrotic cell lesions is impaired by the age-related macular degeneration-associated factor-h his 402 risk variant. J Immunol (2011) 187:4374–83. doi: 10.4049/jimmunol.1002488 21930971

[10] Eisenhardt SU Habersberger J Murphy A Chen Y Woollard KJ Bassler N. Dissociation of pentameric to monomeric c-reactive protein on activated platelets localizes inflammation to atherosclerotic plaques. Circ Res (2009) 105:128–37. doi: 10.1161/CIRCRESAHA.108.190611 19520972

[11] Thiele JR Habersberger J Braig D Schmidt Y Goerendt K Maurer V. Dissociation of pentameric to monomeric c-reactive protein localizes and aggravates inflammation: *In vivo* proof of a powerful proinflammatory mechanism and a new anti-inflammatory strategy. Circulation (2014) 130:35–50. doi: 10.1161/CIRCULATIONAHA.113.007124 24982116

[12] Zhang L Li HY Li W Shen ZY Di WY SR Ji. An ELISA assay for quantifying monomeric c-reactive protein in plasma. Front Immunol (2018) 9:511. doi: 10.3389/FIMMU.2018.00511 29593741 PMC5857914

[13] Molins B Pascual A Llorenç V Zarranz-Ventura J Mesquida M Adán A. C-reactive protein isoforms differentially affect outer blood-retinal barrier integrity and function. Am J Physiol - Cell Physiol (2017) 312:C244–53. doi: 10.1152/ajpcell.00057.2016 28003224

[14] Khreiss T József L Hossain S Chan JSD Potempa LA Filep JG. Loss of pentameric symmetry of c-reactive protein is associated with delayed apoptosis of human neutrophils. J Biol Chem (2002) 277:40775–81. doi: 10.1074/jbc.M205378200 12198121

[15] Khreiss T József L Potempa LA Filep JG. Conformational rearrangement in c-reactive protein is required for proinflammatory actions on human endothelial cells. Circulation (2004) 109:2016–22. doi: 10.1161/01.CIR.0000125527.41598.68 15051635

[16] Molins B Peña E Vilahur G Mendieta C Slevin M Badimon L. C-reactive protein isoforms differ in their effects on thrombus growth. Arterioscler Thromb Vasc Biol (2008) 28:2239–46. doi: 10.1161/ATVBAHA.108.174359 18787187

[17] Romero-Vázquez S Adán A Figueras-Roca M Llorenç V Slevin M Vilahur G. Activation of c-reactive protein proinflammatory phenotype in the blood retinal barrier *in vitro*: Implications for age-related macular degeneration. Aging (Albany NY) (2020) 12:13905–23. doi: 10.18632/aging.103655 PMC7425453 32673285

[18] Schwedler SB Guderian F Dämmrich J Potempa LA Wanner C. Tubular staining of modified c-reactive protein in diabetic chronic kidney disease. Nephrol Dial Transplant (2003) 18:2300–7. doi: 10.1093/NDT/GFG407 14551357

[19] Galván-Román JM Rodríguez-García SC Roy-Vallejo E Marcos-Jiménez A Sánchez-Alonso S Fernández-Díaz C. IL-6 serum levels predict severity and response to tocilizumab in COVID-19: An observational study. J Allergy Clin Immunol (2021) 147:72–80.e8. doi: 10.1016/J.JACI.2020.09.018 33010257 PMC7525244

[20] Zhou F Yu T Du R Fan G Liu Y Liu Z. Clinical course and risk factors for mortality of adult inpatients with COVID-19 in wuhan, China: a retrospective cohort study. Lancet (London England) (2020) 395:1054–62. doi: 10.1016/S0140-6736(20)30566-3 PMC7270627 32171076

[21] Li HY Wang J Wu YX Zhang L Liu ZP Filep JG. Topological localization of monomeric c-reactive protein determines proinflammatory endothelial cell responses. J Biol Chem (2014) 289:14283–90. doi: 10.1074/JBC.M114.555318 PMC4022894 24711458

[22] Gan Q Wong A Zhang Z Na H Tian H Tao Q. Monomeric c-reactive protein induces the cellular pathology of alzheimer’s disease. Alzheimer’s Dement (New York N Y) (2022) 8(1):e12319. doi: 10.1002/TRC2.12319 PMC9270638 35846159

[23] Slevin M Matou-Nasri S Turu M Luque A Rovira N Badimon L. Modified c-reactive protein is expressed by stroke neovessels and is a potent activator of angiogenesis *in vitro* . Brain Pathol (2010) 20:151–65. doi: 10.1111/J.1750-3639.2008.00256.X PMC8094831 19170684

[24] Chirco KR Whitmore SS Wang K Potempa LA Halder JA Stone EM. Monomeric c-reactive protein and inflammation in age-related macular degeneration. J Pathol (2016) 240:173–83. doi: 10.1002/path.4766 PMC5527328 27376713

[25] Fujita C Sakurai Y Yasuda Y Homma R Huang C-L Fujita M. mCRP as a biomarker of adult-onset still’s disease: Quantification of mCRP by ELISA. Front Immunol (2022) 0:938173. doi: 10.3389/FIMMU.2022.938173 PMC9284222 35844576

[26] Munuswamy R De Brandt J Burtin C Derave W Aumann J Spruit MA. Monomeric CRP is elevated in patients with COPD compared to non-COPD control persons. J Inflammation Res (2021) 14:4503–7. doi: 10.2147/JIR.S320659 PMC8434905 34522118

[27] Wang J Tang B Liu X Wu X Wang H Xu D. Increased monomeric CRP levels in acute myocardial infarction: a possible new and specific biomarker for diagnosis and severity assessment of disease. Atherosclerosis (2015) 239:343–9. doi: 10.1016/J.ATHEROSCLEROSIS.2015.01.024 25682033

[28] Karlsson J Wetterö J Weiner M Rönnelid J Fernandez-Botran R Sjöwall C. Associations of c-reactive protein isoforms with systemic lupus erythematosus phenotypes and disease activity. Arthritis Res Ther (2022) 24:139. doi: 10.1186/S13075-022-02831-9 35690780 PMC9188243

[29] Williams RD Moran JA Fryer AA Littlejohn JR Williams HM Greenhough TJ. Monomeric c-reactive protein in serum with markedly elevated CRP levels shares common calcium-dependent ligand binding properties with an *in vitro* dissociated form of c-reactive protein. Front Immunol (2020) 11:115. doi: 10.3389/FIMMU.2020.00115 32117266 PMC7010908

[30] Melnikov I Kozlov S Pogorelova O Tripoten M Khamchieva L Saburova O. The monomeric c-reactive protein level is associated with the increase in carotid plaque number in patients with subclinical carotid atherosclerosis. Front Cardiovasc Med (2022) 9:968267. doi: 10.3389/FCVM.2022.968267 35935662 PMC9353581

[31] Crawford JR Trial JA Nambi V Hoogeveen RC Taffet GE Entman ML. Plasma levels of endothelial microparticles bearing monomeric c-reactive protein are increased in peripheral artery disease. J Cardiovasc Transl Res (2016) 9:184–93. doi: 10.1007/S12265-016-9678-0 PMC4874871 26891844

[32] Li H Gao N Liu C Liu X Wu F Dai N. The cholesterol-binding sequence in monomeric c-reactive protein binds to the SARS-CoV-2 spike receptor-binding domain and blocks interaction with angiotensin-converting enzyme 2. Front Immunol (2022) 13:918731. doi: 10.3389/FIMMU.2022.918731 35874670 PMC9304929

[33] Schumann C Heigl F Rohrbach IJ Sheriff A Wagner L Wagner F. A report on the first 7 sequential patients treated within the c-reactive protein apheresis in COVID (CACOV) registry. Am J Case Rep (2022) 23:e935263. doi: 10.12659/AJCR.935263 35007274 PMC8762613

[34] Zeller J Cheung Tung Shing KS Nero TL McFadyen JD Krippner G Bogner B. A novel phosphocholine-mimetic inhibits a pro-inflammatory conformational change in c-reactive protein. EMBO Mol Med (2022) e16236. doi: 10.15252/EMMM.202216236 36468184 PMC9832874

[35] Zeller J Bogner B McFadyen JD Kiefer J Braig D Pietersz G. Transitional changes in the structure of c-reactive protein create highly pro-inflammatory molecules: Therapeutic implications for cardiovascular diseases. Pharmacol Ther (2022) 235:108165. doi: 10.1016/J.PHARMTHERA.2022.108165 35247517

[36] Fujita C Sakurai Y Yasuda Y Takada Y Huang C-L Fujita M. Anti-monomeric c-reactive protein antibody ameliorates arthritis and nephritis in mice. J Immunol (2021) 207:1755–62. doi: 10.4049/JIMMUNOL.2100349 34470853

