# Corrigendum: C-reactive protein isoforms as prognostic markers of COVID-19 severity

**DOI:** 10.3389/fimmu.2024.1447976

**Published:** 2024-06-21

**Authors:** Blanca Molins, Marc Figueras-Roca, Oliver Valero, Victor Llorens, Sara Romero-Vázquez, Oriol Sibila, Alfredo Adan, Carolina Garcia-Vidal, Alex Soriano

**Affiliations:** ^1^ Group of Ocular Inflammation: Clinical and Experimental Studies, Institut d’Investigacions Biomèdiques Agustí Pi i Sunyer (IDIBAPS), Barcelona, Spain; ^2^ Institut Clínic d’Oftalmologia (ICOF), Hospital Clínic, Barcelona, Spain; ^3^ Statistical Department, Universitat Autònoma de Barcelona, Barcelona, Spain; ^4^ Respiratory Department, Hospital Clinic of Barcelona-IDIBAPS, CIBERES, University of Barcelona, Barcelona, Spain; ^5^ Department of Infectious Diseases, Hospital Clinic of Barcelona-IDIBAPS, University of Barcelona, Barcelona, Spain; ^6^ CIBERINF, Barcelona, Spain

**Keywords:** C-reactive protein, COVID-19, isoforms, inflammation, prognosis

In the published article, there was an error in the Funding statement. The first sentence was incorrectly written as “This work was funded by the Ministry of Science and Innovation of Spain, “Instituto de Salud Carlos III”, “Fondo de Investigación Sanitaria” (grant number PI19/00265), and Funds FEDER “Una manera de hacer Europa”. It should appear as “This study has been funded by Instituto de Salud Carlos III (ISCIII) through the project PI19/00265 and co-funded by the European Union”. The correct Funding statement appears below.

